# Preaching to the choir? Sociodemographic differences in medical students’ evaluation of an undergraduate diversity training module

**DOI:** 10.15694/mep.2018.0000056.1

**Published:** 2018-03-07

**Authors:** Nikki Bruin, Maaike Muntinga, Petra Verdonk

**Affiliations:** 1 VU University; 2 VU University Medical Center

**Keywords:** Diversity, Module evaluation, Student characteristics, Learning outcomes

## Abstract

This article was migrated. The article was marked as recommended.

**Introduction:** Patients with diverse cultural backgrounds experience barriers in access to care, and do not always receive the care they need. To prepare future doctors to provide high quality care for all patients, adequate diversity education is essential. At VUmc School of Medical Sciences, we therefore developed a bachelor second year module on Care ethics and Diversity using an arts-based approach. The aim of this study was to evaluate the module from the students’ perspective, and to gain insight in the relationship between evaluation outcomes and student sociodemographic characteristics.

**Methods:** Design: cross-sectional evaluation study. Module content: after watching three short film fragments, students engaged in a structured dialogue about diversity in relation to care and care giving. Data collection: in 2015 and 2016, a questionnaire containing 12 evaluation items based on module learning outcomes were administered to second year medical students (N=287) immediately after receiving the module. Overall satisfaction with the module was measured using a 1-10 scale (with 1 indicating a very poor, and 10 indicating an excellent evaluation score); learning objective-related items were measured using a 5-point Likert scale. Results were analyzed using multiple linear regression, Pearson’s correlation and Chi-square tests.

**Results:** The average overall satisfaction score was 7.3. We found a strong positive correlation between the overall satisfaction score and the score of the item that measured students’ level of interest (r=.70). Multiple linear regression showed a significant positive effect of identifying as non-native Dutch on multiple evaluation outcomes. Students with non-native Dutch backgrounds reported higher satisfaction with the module, perceived the module as more interesting and more personally relevant, and had higher scores on four of the six learning objective items.

**Discussion:** Our findings indicate that students with non-native Dutch backgrounds were more satisfied with the module and reported more learning than their native Dutch peers. This might be explained by the fact that diversity education acknowledges Dutch students’ lived experiences and builds on preexisting embodied diversity knowledge. Conversely, (white) native Dutch students might not always perceive diversity knowledge as legitimate or consider diversity as something that does not concern them.

**Conclusion:** Non-native Dutch students reported more overall satisfaction with diversity education and more diversity learning than majority students. To secure and advance high quality health care for all patients, medical schools should invest in researching and developing diversity content that engages all students.

## Introduction

Health care professionals working in Western medical systems have been confronted with an increasingly diverse patient population. This diversity comes with differences in health beliefs, health behaviors and practices, and attitudes towards health care providers [1]. As a result, physicians are expected to provide high quality care to patients with varying needs and expectations, and safeguard equitable access to diagnosis, treatment and services. Equitable access has been defined as ‘care that does not vary in quality because of personal characteristics, such as gender, ethnicity, geographical location and socioeconomic status’ [2]. However, research has shown that patients with non-majority backgrounds often experience a lower quality of care and poorer access, indicating that the current medical workforce is inadequately meeting the demands of a rapidly diversifying society [ 2- 4].

One of the possible explanations for the disparities between majority and non-majority patients is a lack of diversity-responsiveness at a health system and individual health worker level. Diversity-responsive institutions and professionals adequately recognize and respond to key cultural features that affect clinical health [1; 5]. Such an adequate response is crucial, because cultural differences and language barriers between doctors and patients influence the successful delivery of health care [2]. In addition, limited access to diversity responsive care results in delayed entry into the health care system. Such barriers do not only stand in the way of effective health care, but are also problematic from a value-based perspective: equitable access to care is a human right. Actively facilitating marginalized groups in the pursuit and enactment of this right is a core responsibility of a socially just medical system and society [1], and should therefore be sustainably integrated in both graduate and undergraduate education [ 6- 10].

To prepare future doctors for a working environment where providing diversity responsive care is a norm and a necessity, the Amsterdam, the Netherlands-based VUmc School of Medical Sciences (VUmc) has been implementing an educational track ‘Interculturalization and Diversity’ into the undergraduate curriculum [11]. The track is rooted in the Dutch Blueprint for final objectives of medical education, which addresses several CanMeds competencies [12; 13], and content aligns with diversity learning objectives related to knowledge, communicative skills and reflective skills [11]. Educational material developed within the educational track is based on a critical notion of diversity that goes beyond the three ‘master categories’ gender, race/ethnicity and class, and includes additional categories of difference (such as sexual identity, migrant status and language) and their intersections. Throughout the track’s design and development, medical education professionals and a performance and visual artist collaborated to explore teaching approaches that connect diversity and identity to the idea of the self instead of the other, with the aim to increase students’ empathy and critical consciousness [11]. Implementation of the track has been receiving comprehensive support from key stakeholders in the organization, which reflects a shared institutional consciousness of the importance of fixing the knowledge, the numbers and the institution for an equitable and inclusive health care system [11].

### The module Care Ethics and Diversity

One of the track’s products that has recently been implemented in the undergraduate curriculum is the bachelor 2 arts-based module ‘Care ethics and Diversity’, designed for a small group teaching setting. The two-hour module takes place during the course ‘Physician & Patient 3’, which mainly centers on medically and socially complex problems. The module’s learning objectives are as follows: (1) students have knowledge of the concepts culture, medical culture, in- and exclusion in health care, discrimination/racism and stigma; (2) students understand the role of language barriers in relation to in- and exclusion in health care; (3) students have knowledge of care ethics and can recognize the phases of care in (cross-cultural) care practices; (4) student can reflect in their own role in managing different expectations of care professionals and the health care system. All module instructors are affiliated with the department of Medical Humanities at VUmc, but are of different academic backgrounds (e.g. psychology, sociology, medicine and cultural anthropology). The module was developed in close collaboration with a performance artist.

The aim of the module is to address diversity among VUmc physicians, to encourage students to reflect on their own background in relation to (future) experiences on the clinical work floor, and to contribute to the visibility of role models with various cultural backgrounds. Although role models are important for acquiring skills related to ethics and diversity [14], students seldom encounter socioculturally diverse role during their time in medical school. In addition, culturally diverse students bring important experiential knowledge into the classroom and clinical workplace, knowledge which is often overlooked and undervalued. The module ‘Care ethics and Diversity’ acknowledges the legitimacy of such knowledge by - literally - visualizing its professional relevance using film fragments.

Module content consists of a theoretical part, three film fragments (developed by the collaborating artist) and a classroom dialogue. Film as a method was chosen because organizing the presence of physicians with diverse backgrounds at all small group sessions was logistically challenging. The module is taught as follows: first, students receive theoretical and conceptual input (e.g., four phases of care [15], definition of stigma and discrimination). Subsequently, the film fragments are shown. The fragments show three VUmc physicians with various cultural backgrounds (Turkish-Flemish, Afghan-Dutch, and native Dutch) sharing accounts of how their cultural background or migration history influences their daily practices and experiences. For instance, the physician with a Turkish-Flemish background presents a dilemma he faced when confronted with a Turkish patient’s wish to not share a terminal diagnosis with his wife. “What would *you* do?”, the physician in the film asks the students after sharing his story. Students are then encouraged to analyze the physician’s dilemma from a care ethics point of view, explore their own and each other’s’ perspectives related to the dilemma, and reflect on how their own background plays a role in their individual analysis of the situation.

The module ‘Care ethics and Diversity’ is an example of an arts-based intervention. For several decades, art forms such as film and theater have been used as a tool to teach social, political and emotional issues (i.e., professional development) in medicine in different professional fields [ 16- 18], for instance in medical ethics education [19]. Recently, arts-based interventions are increasingly used to teach medical and other health sciences students about diversity issues in medicine [20; 21]. Although the impact of film and other arts-based interventions on engagement and learning has been topic of investigation - within as well as outside of health sciences education - more insight is needed in how medical students evaluate arts-based diversity education and how evaluation outcomes between students differ. Such insight helps to optimize the impact of diversity education for all learners [16; 20].

### Study objective and research question

We therefore carried out a module evaluation. Aim of the evaluation was to assess whether the learning objectives were accomplished, and to further adjust the module content to the learning needs of students. We asked the following research question: “How do undergraduate medical students evaluate the second year module “Care ethics and Diversity, en to what extent do evaluation outcomes differ between social groups?” Research activities included an investigation of students’ overall satisfaction with the module, an assessment of the correlation between students’ overall satisfaction with the module and the extent to which they perceived the learning objectives accomplished, and a calculation of the relationship between evaluation outcomes and students’ sociocultural characteristics. We aim to gain more insight in the way in which undergraduate medical students evaluate arts-based diversity education, and to identify student characteristics-related differences in evaluation outcomes. In doing so, we hope to contribute to the increasing knowledge about optimizing diversity learning and teaching in health professions education.

## Methods

### Design and setting

We used cross-sectional evaluation data to answer our research question. The study took place at the VU University Medical Center School of Medical Sciences (VUmc SMS) in Amsterdam, the Netherlands. VUmc SMS has a diverse student population. In 2009-2010,20% had a non-native Dutch background [22]. Organizational and curricular diversity-responsiveness is a key VUmc SMS policy aim, and support for diversity related educational initiatives exists at both an institutional and staff level [11].

### Study population and sample

We included all second year undergraduate students who took the module during the academic year 2015-2016 and 2016-2017. Five hundred students filled out the evaluation questionnaire. After removing all incomplete questionnaires (i.e., missings on all sociodemographic items) from the analyses (N=213), our final study sample was 287 (57.4%). The high amount of incomplete questionnaires is likely a result of the lay out of the paper questionnaire (sociodemographic items were on the back side).

### Data collection and outcome measures

A 12-item Dutch-language evaluation questionnaire was administered by the instructors immediately at the end of the module. Written consent was obtained on the questionnaires.

*Overall satisfaction* with the module was measured on a 1-10 scale (1=minimum satisfaction, 10=maximum satisfaction).

*Perceived extent to which the module was interesting, perceived professional relevance* and *perceived personal relevance* were measured using a 5-point Likert scale (1=totally disagree, 5=totally agree). Students were asked to what extent they agreed with the following statements: “The module care ethics and diversity is interesting”, “The module care ethics and diversity is relevant for my future professional practice”, “The module care ethics and diversity is relevant for me personally”.

Items related to the module’s learning objectives were measured using a 5-point Likert scale (1=totally disagree, 5=totally agree). Students were asked to what extent they agreed with a statement related to each of the learning objectives. The items included: (1) *perceived increase of awareness of own diversity-related norms* (“The module care ethics and diversity has increased my awareness of my own diversity-related norms and values”); (2) *perceived increase of awareness of own norms about good care* (“The module care ethics and diversity has increased my awareness of my own norms and values about ‘good care’”); (3) *perceived learning about in- and exclusion within the health care system* (“The module care ethics and diversity has taught me about in-and exclusion within the health care system”); (4) *perceived learning about language barriers in the health system* (“The module care ethics and diversity has taught me about the role of language barriers in health care”); (5) *perceived increase in learning about managing different expectations from patients* (“The module care ethics and diversity has taught me about managing differences in patient expectations of care professionals”), and (6) *perceived increase of awareness of diversity among doctors and colleagues* (“The module care ethics and diversity has increased my awareness of diversity among physicians and colleagues’).

Sociodemographic variables included sex (male/female/other), age, ethnic background (native Dutch/non-native Dutch), county of birth mother (Netherlands/other), country of birth father (Netherlands/other); religious background (religious/nonreligious); current living situation (living at home/living with roommates/living alone/living with a partner or family); country of growing up (the Netherlands/other); living environment growing up (village/small city/large city/other).

Ethnic background and religious background were measured using self-identification with string variables, but were recoded into dichotomous variables for analytical purposes because of large response variations. The string variables *Nederlands* and *Hollands* (both indicating a native Dutch cultural background) were recoded into ‘native Dutch background’. The string variables Afghan, African, Arab, Belgian, Bengali, Dutch-British, Chinese, Dutch-Ethiopian, (Dutch-)Egyptian, (Dutch-) German, Ghanaian, Dutch-Greek, Hindustani, Indonesian, Iranian, Iraqi, Kurdish, Latino, (Dutch-, Surinamese -, Spanish-)Moroccan, Dutch-Nigerian, non-Western, Pakistani, Dutch-Peruvian, (Dutch-) Surinamese, Somalian, Syrian, South-American, and (Dutch-)Turkish were recoded into ‘non-native Dutch background’. The string variables ‘agnostic’, ‘atheist’, ‘existentialist’, ‘none’, ‘not-religious’, ‘non-believer’, and ‘nothing’ were recoded into ‘nonreligious background’. The string variables Christianity/Christian, Protestantism/Protestant, Lutheran, Reformed, Hindu/Hinduism, Islam/Muslim and (Roman)Catholic/Catholicism were recoded into ‘religious background’.

### Statistical analysis

Data was analyzed using SPSS, version 24. Chi-square tests were used to assess whether there was a significant difference between the 2015-2016 and 2016-2017 cohort for sociodemographic characteristics. Pearson’s r was calculated to investigate the inter-item correlation between the overall satisfaction item score and the individual learning objective-related item scores.

We used multiple linear regression to assess the relationship between the overall satisfaction item score and sociodemographic variables, and between the learning objective-related item scores and sociodemographic variables. A correlation matrix was computed of all the dependent and independent variables to check for multicollinearity. All sociodemographic characteristics were added to the model. Dummy variables were computed for the categorical variables. Linearity of the dependent variable was assumed because the study population was bigger than N=30. Effect modification was analyzed by computing interaction terms. ANOVA was calculated to assess model fit.

## Results

Chi-square tests showed a small but significant difference between Cohort 1 (n=172) and Cohort 2 (n=115) in terms of ethnic background, country of birth father, country of birth mother, religious background and country of growing up. However, to increase power, the cohorts were aggregated for further analysis.

Students’ mean age was 20, and 75 % was female. A total of 29% of students identified as having a non-native Dutch background, while 11% was born outside of the Netherlands and 9% had not grown up in the Netherlands. Most students identified as having a religious background (40%), 28% of the students reported having a nonreligious background. Most students lived alone (47%) or with their parents (41%). The average overall module satisfaction score was 7.3 (range 2-9). Highest scores were given for the item ‘relevance for future professional practice’. Students reported learning most about diversity among doctors and colleagues. See Table 1 for all results.

**Table 1.** Sociodemographic characteristics and evaluation outcomes of the study population (N=287).

Variable	
*Age (mean(range))*	20 (18-35)
*Gender N(%)* Female	214 (74.6)
*Ethnic background N(%)* Native Dutch Non-native Dutch	203 (70.7) 87 (29.3)
*Country of birth N(%)* Netherlands Other	255 (88.9)32 (11.1)
*Country of birth father N(%)* Netherlands Other	211 (73.5) 76 (26.5)
*Country of birth mother N(%)* Netherlands Other	207 (72.1) 80 (27.9)
*Religious background N(%)* Religious Nonreligious Missing	115 (40.1) 109 (38.0) 63 (22.0)
*Country of growing up N(%)* Netherlands Other	261 (91.0) 26 (9.1)
*Living environment growing up N(%)* Village mall city Big city Other	94 (32.8) 96 (33.4) 85 (29.6) 12 (4.2)
*Living situation N(%)* With parents Alone With roommates With family/a partner	118 (41.1) 135 (47.0) 25 (8.7) 9 (3.2)
*Evaluation outcomes (mean (range))* Overall module satisfaction Extent to which the module was interesting Professional relevance Personal relevance Learning objectives Increased awareness of own diversity-related norms Increased awareness of own norms about good care Learned about in- and exclusion within the health care system Learned about language barriers in the health system Learned about managing different expectations Increased awareness of diversity among doctors and colleagues	7.3 (2-9) 3.95 (2-5) 4.12 (1-5) 3.79 (1-5) 3.49 (1-5) 3.69 (1-5) 3.67 (1-5) 3.50 (1-5) 3.80 (1-5) 3.88 (1-5)

### Inter-item correlation

We found significant correlations between all learning objective scores and the overall module satisfaction score (r= .33 - .47). A high correlation was found between the overall satisfaction score and the item ‘perceived extent to which the model was interesting’ (r= .70, n= 287, p=.00 [23]).

### Linear regression

Multicollinearity was detected between the sociodemographic variables ‘country of birth father’ (r= .81) and ‘country of birth mother’ and ‘ethnic background’ (r= .81 and r=.73 respectively) [23]. To adhere to the assumptions of the use of linear regression analysis, these variables were removed. As shown in Table 2, the ANOVA model fit is not significant for all models. Whilst correcting for this via a backwards procedure, the model became significant but the effects of the sociodemographic characteristics became smaller. Correcting did not result in a change in significance of the independent variables. To present the full effects the complete, but insignificant model is presented.

Multiple linear regression showed a significant positive effect of identifying as non-native Dutch on multiple evaluation outcomes. Students with non-native Dutch backgrounds had higher average overall satisfaction scores, and had higher average scores on the items ‘Perceived extent to which the module was interesting’ and ‘perceived personal relevance’, and scored higher on four of the six learning objective items. Male gender was negatively associated with ‘increased awareness of students’ own norms about good care’, as was having a religious background and having grown up in a city. Students who lived with roommates were more likely to find the module interesting, but less likely to report having learned about managing expectations of different patients. Students who lived alone were less likely to report having learned about diversity among doctors and colleagues. See Table 2 for all results.

**Table 2.** Multiple linear regression for the relation between the evaluation item scores and the sociodemographic characteristics of the study population (N=287). Only the independent variables with significant P-values are presented.

	Regression			ANOVA model fit
Model	Beta	95% CI	p-value	sig.
*Overall satisfaction*	7.13			.18
Ethnic background	.26	.16 - .88	.01	
Country of birth	-.11	-.84 - .19	.02	
*Extent to which the module was interesting*	3.87			.25
Ethnic background	.22	.06 - .56	.02	
Living situation: with roommates				
.15	.01 - .41	.04	
*Personal relevance*	3.69			.31
Ethnic background	.26	.16 - .84	.00	
*Increased awareness of own norms about good care*	3.93			.01
Gender	-.14	-.45 --.01	.04	
Ethnic background	.26	.14 - .71	.00	
Religious background	-.24	-.57 - -.14	.00	
Living environment growing up: small city	-.18	-.55 - -.03	.03	
Living environment growing up: big city	-.18	-.52 - -.03	.03	
*Increased awareness of in-and exclusion*	3.66			.36
Country of growing up	.16	.01 - .93	.05	
*Increased learning about managing different patient expectations*	3.72			.07
Living situation: with roommates	-.17	-.10 - .31	.02	
*Increased awareness of diversity among doctors and colleagues*	3.88			.15
Ethnic background	.18	.01 - .56	.05	
Living situation: alone	-.15	-.75 - -.02	.04	

## Discussion

The objective of this study was to assess how medical students evaluated the second year module ‘‘Care ethics and Diversity’’, and the extent to which evaluation outcomes were associated with students’ sociodemographic characteristics.

Our results show a high correlation between overall student satisfaction and the extent to which students considered the module to be interesting. This might indicate that the overall satisfaction score is primarily a reflection of students’ idea of the module’s content as engaging and, perhaps, entertaining, and less of students’ evaluation of the module’s personal or professional relevance or of the extent to which learning had taken place. Although student engagement is an important prerequisite for learning, our findings warrant a closer look at the validity of our current evaluation constructs, and their usefulness for the assessment of the impact of our educational track. While attempts to measure (changes in) cross-cultural competencies (such as communication skills and knowledge about social determinants of health) are ubiquitous and have been elaborated on in the literature [5; 24-26], we envision diversity-responsiveness of medical professionals and the health care system to go beyond knowledge and skills, but to also include critical analytical skills (the ability to contextualize phenomena and connect them to theory) and a consciousness of power and privilege in relation to the self [27]. The latter competencies are challenging to measure with standard quantitative approaches, because in most individuals, diversity-awareness is an evolution rather than a revolution, and its advancement involves on-going educational input and exposure [28]. Mixed method or qualitative evaluation methods, such as participatory research or arts-based approaches, might be better equipped to capture the complexity of diversity learning and doing [20; 29].

The outcomes of the multiple linear regression showed that non-Dutch ethnic background was positively associated with higher scores on multiple evaluation outcomes. Students who identified as non-native Dutch reported higher overall satisfaction with the module, were more likely to perceive the module as interesting and to consider the content personally relevant, and reported more learning related to the module’s six learning objectives than students who identified as native Dutch. These outcomes suggest differences in the level of learning that takes place during diversity education, and in the way in which students with non-native Dutch and native Dutch background experience modules with diversity content at VUmc SMS.

Previous research has provided insight in the impact of diversity education on diversity learning and consciousness. For instance, there is evidence that the relationship between the amount of training received and training outcomes is not necessarily linear [24], that training effects do not always sustain [20] and that training does not always translate to more diversity aware or culturally sensitive medical students or professionals. For instance, Beagan (2003) reported that, after the introduction of a new course addressing social and cultural issues in medicine, students’ awareness of such issues had not always improved - contrary to expectation, students in some cases even denied social inequalities and their own privilege [30]. Seeleman et al. found a discrepancy between self-perceived diversity competence and actual competencies [26]. Differences in diversity learning outcomes between learners based on sociocultural background has previously been reported as well. Beagan found that students belonging to a minority or marginalized group were more likely to believe that membership of said group had an effect on the physician-patient interaction [30]. Ivory et al. evaluated the outcomes of a theater-based workshop centering on teaching students about the effects of marginalization and culture on health outcomes and found that women and students with no past diversity training reported most positive changes in terms of perceptions and attitudes [20]. The above examples show that an across-the-board positive impact of diversity education is far from self-evident.

### Different diversity learners

A lack of situational interest in diversity as a learning topic might be at the root of the differences in evaluation outcomes between non-native Dutch and Dutch students [31]. Previous research in the experiences of students with non-native Dutch backgrounds at VUmc SMS suggests that these students deal with exclusionary, ‘othering’ practices such as micro-aggressions and stereotyping throughout their medical school career [22; 32; 33]. Because power structures within institutions of higher education mirror broader sociocultural hierarchies, non-native Dutch students often also experience exclusion in their daily life. In the Netherlands, non-white individuals with migrant backgrounds are generally considered ‘not really Dutch’ and experience institutionalized, cultural and personal racism based on a perpetual minority status [34]. Lived experiences of being the cultural or political ‘Other’ might translate into embodied knowledge about the role aspects of diversity (such as ethnicity, race, language and religion) play in health and healthcare. For instance, students might have insight in the impact of language barriers on health because they have interpreted for a family member during a doctor’s visit. Students who carry their experiences with them into the classroom might therefore already have a diversity-related knowledge base, which benefits deep learning [31]. Moreover, they might be more likely to value diversity content as relevant, urgent and legitimate, which is related to situational interest and increases intrinsic motivation to learn [6; 10; 35]. Preexisting embodied diversity knowledge, therefore, is likely to translate into student activation and engagement with diversity content.

Conversely, absence of such knowledge might result in less engagement - or even disengagement - in native Dutch students. Research into the experiences of medical students with diversity curricula suggests that native Dutch students’ learning might be impeded by the notions that diversity learning does not concern them, is medically irrelevant, or obsolete [32; 36]. Zembylas and colleagues adapted Tronto’s idea of ‘privileged irresponsibility’ to the educational environment [37]. Privileged irresponsibility refers to the way in which a majority group fails to acknowledge power dynamics at a sociostructural level, because their membership to the dominant social group guarantees that they are unaffected by the consequences of an imbalance in these dynamics. The ability of white students to deny the existence of racialized or ethicized oppression (and how it benefits them) can, for instance, result in the claim to be ‘color blind’ [38]. This claim inherently delegitimizes diversity issues that go beyond biostatistical, genetic or cultural knowledge, absolving students from critically reflecting on their own social position and taking responsibility for the needs of others. As Sarah Ahmed argues, this imposes on non-majority - often non-white -bodies the work of not only educating their majority peers about diversity, but also taking emotional and intellectual responsibility for non-majority patients and colleagues [39]. To further understand how differences in diversity learning are constructed and how learning can be optimized for all students, insight in how whiteness (or ‘Dutchness’) is performed by medical students during diversity education in VUmc SMS is essential.

### Strengths and limitations

This study has several strengths, which include the large sample, the ability to correct for multiple confounders and reduction of selection bias resulting from the fact that all the students were asked to fill out the evaluation form. Recall bias was limited due to the fact that the evaluations were filled out immediately at the end of the module. Another strength was the use of self-identification to measure ethnic background. Measuring ethnicity using predefined categories is problematic, because it increases the chance that a respondent reports within a category they do not fully identify with [40].

This evaluation study also has limitations. We relied on students’ self-reports to measure learning and increased awareness, which challenges an evaluation of ‘actual change’. Low scores on learning objective items might reflect a low level of perceived learning (e.g., in disengaged students, or in students who felt that the module did not teach them anything new), but might paradoxically also reflect a high level of perceived learning (e.g., in students who during the module became aware of their own incompetence [41]). For instance, both male students and students with religious backgrounds reported lover scores on the item that aimed to measure whether the module had contributed to students’ awareness of their own normative frameworks related to ‘good care’. Our data cannot relate either group to either a lack of awareness or to preexisting high awareness, and explanations for our findings remain purely theoretical. In the same vein, high scores might reflect both high and low levels of perceived learning (e.g., in students who were unaware of their own incompetence). Our finding that students with non-native Dutch background persistently had higher scores across the evaluation items (both on the overall satisfaction and learning objectives items) points to actual differences in perceived learning between groups.

Furthermore, by aiming to measure the effect of the module, we assumed linearity between diversity education and increased learning about diversity - an assumption which, as we addressed previously in this article, may not be correct. Finally, the overall satisfaction score was theorized to capture a total experience of the module, including whether the module was interesting and relevant and whether the learning objectives had been achieved. However, results of our inter-item correlation calculations suggest that the overall satisfaction score is more likely to be primarily a measurement of level of interest (or ‘entertainment’). Future student evaluations might employ more longitudinal designs to increase insight in the relationship between diversity education and diversity learning in medical education [24; 25; 29].

## Conclusion

Our findings reveal that students with non-native Dutch backgrounds are more satisfied with diversity content and report more learning than their native Dutch peers, which confirms the added value of subgroup analysis in diversity education evaluation. Because diversity learning is an ongoing process and the impact of training on consciousness, attitudes and skills might not be a linear one, one training module does not suffice [28]. To secure high quality care for patients of all backgrounds, it is essential that students are exposed to diversity teaching throughout their medical school careers, and that all students engage with diversity content with equal intent and attentiveness. Although health professions curricula should aim to target differences in learning outcomes by creating diversity content that stimulates situational interest in students who lack personal interest in diversity, we as medical educators should also be mindful not to rely on peers with embodied diversity knowledge to do the hard work for us. An investigation of how ‘whiteness’ or ‘native Dutchness’ is enacted during diversity education can help us gain insight in ways to avoid ‘preaching to the choir’, and maximize diversity learning for all.

## Ethics

Under Dutch legislation, no ethical permission is required for this type of study as the evaluation does not involve an intervention, but a module that is part of a regular education program all students are required to participate in. Module or course evaluations such as the one described in this study are common practice in higher education in the Netherlands. Participation in the study was not compulsory, and students who chose to participate filled out an informed consent form. This article study follows the principles of the Declaration of Helsinki.

## Take Home Messages

• Investigating the relationship between student sociocultural characteristics and student evaluations of diversity content reveals significant differences in satisfaction and learning between students of majority and non-majority background.
• Quantitative measurements might not be adequately capable of assessing the impact of diversity teaching on diversity learning and on processes related to building critical consciousness.
• An investigation of how majority privilege plays a role in diversity learning of medical students is necessary to optimize diversity teaching for all learners.

## Notes On Contributors

Nikki Bruin, BSc, received her Bachelor’s Degree in Health Sciences from the VU University in Amsterdam, the Netherlands.

Maaike Muntinga, PhD, is a postdoctoral researcher at the department of Medical Humanitites at VU University Medical Center in Amsterdam, the Netherlands.

Petra Verdonk, PhD, is an associate professor at the department of Medical Humanitites at VU University Medical Center in Amsterdam, the Netherlands.

## Declarations

Dr Petra Verdonk declares a conflict of interest as a current guest theme co-editor of “Diversity in Medical Education” theme in AMEE MedEdPublish. There are no other conflicts of interest to declare.
