# Supplementary material for: Preaching to the choir? Sociodemographic differences in medical students’ evaluation of an undergraduate diversity training module
Source: MedEdPublish (2016). 2018 Mar 7;7:56. [Version 1] doi: 10.15694/mep.2018.0000056.1 (PMC10711956; doi:10.15694/mep.2018.0000056.1)
Supplement: Peer review history

# Reviewer report: Reviewer response for version 1

**DOI:** 10.21956/mep.19529.r28907

**Published:** 2018-04-16

**Authors:** Rashmi Kusurkar

**Affiliations:** 1 Amsterdam UMC, Faculty of Medicine Vrije University Amsterdam

This review has been migrated. The reviewer awarded 5 stars out of 5 I think this paper uncovers an important problem: ethnic majority students find diversity-related educational modules less relevant that ethnic minority students. If students are not convinced about the relevance of what they are learning, they are unable to internalize this learning as a value. The teaching then fails to autonomously motivate them to change their behaviour when they deal with diversity in their clinical practice. (1) If we want change in behaviour, we need to intrinsically/autonomously motivate our students for learning. Reference:(1) Ten Cate TJ, Kusurkar RA, Williams GC. How can self-determination theory assist our understanding of teaching and learning processes in medical education. AMEE Guide 59. Medical Teacher 2011; 33: 961-973.

Reviewer Expertise:

NA

No decision status is available

# Reviewer report: Reviewer response for version 1

**DOI:** 10.21956/mep.19529.r28906

**Published:** 2018-03-26

**Authors:** Janusz Janczukowicz

**Affiliations:** 1 Medical University of Lodz

This review has been migrated. The reviewer awarded 5 stars out of 5 This is a very interesting paper presenting the complexities of the Dutch educational context that superficially might be perceived as free of racism and “colour blind” (quoting Wekker). The question is how to address the fact that white-native-Dutch students may not perceive diversity as something important and worth learning and what educational interventions should be developed to increase their involvement. Another interesting aspect of this paper is the description of using the art-based approach to the diversity- related module. The role of humanities in medical education is currently being re-discovered as the perfect tool for enhancing self-reflection and developing patient-centred sensitivity and empathy.

Reviewer Expertise:

NA

No decision status is available

# Reviewer report: Reviewer response for version 1

**DOI:** 10.21956/mep.19529.r28908

**Published:** 2018-03-23

**Authors:** Trevor Gibbs

**Affiliations:** 1 AMEE

This review has been migrated. The reviewer awarded 5 stars out of 5 A very interesting paper to read and a very important one to add to the collection on diversity. It is perhaps surprising how ones initial thoughts are clouded by unfounded perceptions- as a UK citizen I would always look to the Dutch for their tolerance and support of diversity. however it looks like numbers perhaps take over hearThe other thought that I took away from this paper was the need to have some sort of constancy regarding certain issues throughout the curriculum- almost a golden thread running through the curriculum- diversity should be one of those golden threads

Reviewer Expertise:

NA

No decision status is available
